# Muscle Weakness: A Misleading Presentation in Children With Distinctive Syndromic Entities (Clinical Case Reports)

**DOI:** 10.1177/2324709616689583

**Published:** 2017-01-01

**Authors:** Ali Al Kaissi, Sergey Ryabykh, Polina Ochirova, Vladimir Kenis, Jochen G. Hofstätter, Franz Grill, Rudolf Ganger, Susanne Gerit Kircher

**Affiliations:** 1 Hanusch Hospital, Vienna, Austria; 2 Orthopedic Hospital of Speising, Vienna, Austria; 3 Iliazarov Center, Kurgan, Russian Federation; 4 Pediatric Orthopedic Institute, Saint-Petersburg, Russia; 5 Center of Pathobiochemistry and Genetics, Medical University of Vienna, Vienna, Austria

**Keywords:** myopathy, progressive pseudorheumatoid arthritis, WISP 3 gene mutation, *47*,XXY (aneuploidy of Klinefelter syndrome), mutations in the N-acetylgalactosamine-sulfate sulfatase gene (GALNS gene)

## Abstract

Marked ligamentous hyperlaxity and muscle weakness/wasting associated with awkward gait are the main deficits confused with the diagnosis of myopathy. Seven children (6 boys and 1 girl with an average age of 8 years) were referred to our department because of diverse forms of skeletal abnormalities. No definitive diagnosis was made, and all underwent a series of sophisticated investigations in other institutes in favor of myopathy. We applied our methodology through the clinical and radiographic phenotypes followed by targeted genotypic confirmation. Three children (2 boys and 1 girl) were compatible with the diagnosis of progressive pseudorheumatoid chondrodysplasia. The genetic mutation was correlated with the *WISP 3* gene actively expressed by articular chondrocytes and located on chromosome 6. Klinefelter syndrome was the diagnosis in 2 boys. Karyotyping confirmed *47*,XXY (aneuploidy of Klinefelter syndrome). And 2 boys were finally diagnosed with Morquio syndrome (MPS type IV A) as both showed missense mutations in the N-acetylgalactosamine-sulfate sulfatase gene. Misdiagnosis can lead to the initiation of a long list of sophisticated investigations.

## Introduction

The differential diagnosis of myopathy is broad and includes conditions that present with abnormal gait, muscle weakness, and serum creatine kinase elevation or any combination of these findings. Several etiologies should be considered and excluded in patients with progressive muscle weakness such as myotonic dystrophies, metabolic (glycogenosis, lipidosis, and mitochondrial defects), and we should also consider sarcoid and amyloid-associated myopathies.^1,2^

Spranger et al^3^ first described a progressive pseudorheumatoid (chondro-)dysplasia (PPD or PPAC) as a progressive connective tissue disease, which combined the radiological features of Scheuermann’s disease, with radiographic features of juvenile rheumatoid arthritis. The disorder is classified as an autosomal, recessively inherited chondrodysplasia, with absence of inflammatory parameters. Pain, swellings, and stiffness of the joints are characteristics; the findings are mistaken for juvenile rheumatoid arthritis, although with absence inflammatory parameters.^4^

Klinefelter syndrome is defined as a group of chromosomal disorders in which there is at least one extra X chromosome compared with the normal 46,XY male karyotype. Tall, thin-built males with eunuchoid appearance and a slightly underdeveloped penis and gynecomastia with mild mental retardation are the main characteristics of Klinefelter syndrome.^5,6^

Morquio’s syndrome is an autosomal recessive dysplasia caused by a deficiency in the enzyme N-acetylgalactosamine-sulfate sulfatase gene (GALNS gene), which is essential for the degradation of keratan sulfate and chondroitin-6-sulfate on chromosome 16q24.^7,8^

The diagnosis of most musculoskeletal disorders in children should be based on clinical grounds and must be interpreted in the clinical context of the disease. As laboratory tests may not be diagnostic, and delay in the diagnosis of various forms of musculoskeletal pathology can adversely affect the outcome.^9,10^

The value of this article is 5-fold; first, when myopathy was suspected, this was followed by a series of sophisticated investigations, none of these precisely defined the disorder. Second, hypotonia, ligamentous hyperlaxity, and waddling gait are misleading presentations. Third, the progressive nature of arthropathy was the incentive for radiographic documentation to rule out myopathy and confirmed the diagnosis of progressive pseudorheumatoid arthropathy of childhood (PPAC). Fourth, the clinical and the radiographic phenotypes in Klinefelter (XXY) and in Morquio’s syndromes are characteristic and somehow pathognomonic. Fifth, physicians caring for children should be capable of assessing the musculoskeletal system in correlation with clinical and radiographic phenotypes.

## Materials and Methods

The study protocol was approved by the Ethics Committee of the Turner Scientific Research Institute (No. 3/2016), Saint-Petersburg, Russia); in addition, informed consent was obtained from the patients’ guardians. Seven children (6 boys and 1 girl with an average age of 8 years) of different ethnic origins were enrolled at the Osteogenetic Department of the Orthopaedic Hospital of Speising, Vienna, Austria; Axial Skeleton and Neurosurgery Department, Ilizarov Center, Russia; and the Turner Institute of Children Orthopedics, Saint-Petersburg, Russia.

The subsequent developmental history in all children was almost normal until the age of 2 to 3 years.

We subdivided our patients in accordance with the muscle weakness and ligamentous stiffness and/or hyperlaxity into 2 groups.

### Group 1

Muscle weakness was associated with stiff joints and gait. Difficulties from 3 years of age onwards was a prominent feature. Mild proximal and distal (legs, arms, and axial) weakness accompanied with moderate articular stiffness and mild flexion contractures of the interphalangeal joints and tightness of the hamstrings and tendo Achilles were the most prominent features in 3 children. Diffuse arthralgia with progressive joint enlargement over the elbows, wrists, fingers, knees, and ankles were notable features. Back pain was a uniform clinical presentation.

From the motor perspective, there were increasing difficulties, and the distance that our patients were able to walk had decelerated. Later in life, these children manifested enlarged joints with functional limitations (coxalgia) resembling patients with rheumatoid arthritis.

#### Investigations

Serum creatine kinase and plasma lactate were normal. Electromyography showed minimal myopathic changes, though past muscle magnetic resonance imaging (MRI) showed minimal, nonspecific, and nondiagnostic changes. Muscle biopsy and muscle respiratory chain were normal as well. Selenoprotein-related myopathy (SEPN1) and ryanodine receptor 1 (RYR1) gene-related myopathy were performed in one patient and showed no mutations. Later on, the progressive osseous expansion of the phalanges was the reason to suspect the diagnosis of juvenile rheumatoid arthritis. But the negative laboratory results of juvenile rheumatoid arthritis made the diagnosis invalid.

### Group 2

Muscle weakness associated with joint hyperlaxity and gait difficulties from 2 years of age onwards was a prominent feature in 3 children. The prime clinical presentation in this group was weak muscles in all patients, hypotonic muscles. Two boys aged 9 and 10 years presented with tall stature (97th percentile). Thin built with increased limb growth associated with eunuchoid appearance, and both manifested slightly underdeveloped penis, small testicles, and mild gynecomastia. Endocrinopathy was ruled out, and hypothyroidism, hyperthyroidism, hyperparathyroidism, and vitamin D deficiency were negative; in addition, they showed normal inflammatory markers. Clinically, they did not manifest systemic rash, fever, lymphadenopathy, and/or sacro-iliitis or any ophthalmological or internal organ involvement. Laboratory investigations were undertaken and included full blood count, erythrocyte sedimentation rate, C-reactive protein, rheumatoid facto, antinuclear antibodies, and HLA-B27, all of which showed normal results.

#### Investigations

Serum creatine kinase and plasma lactate were normal. Electromyography showed minimal myopathic changes, though past muscle MRI showed minimal, nonspecific, and nondiagnostic changes. In addition, SEPN1 and RYR1 gene-related myopathy were performed in one patient and showed no mutations. The muscle biopsies and the overall investigations were nonspecific and noncompatible with myopathy.

## Results

### Group 1

The skeletal survey, including lateral skull, spine, hands, and pelvis, were performed in our center and the images were compatible with progressive PPAC.

**Figure 1.**
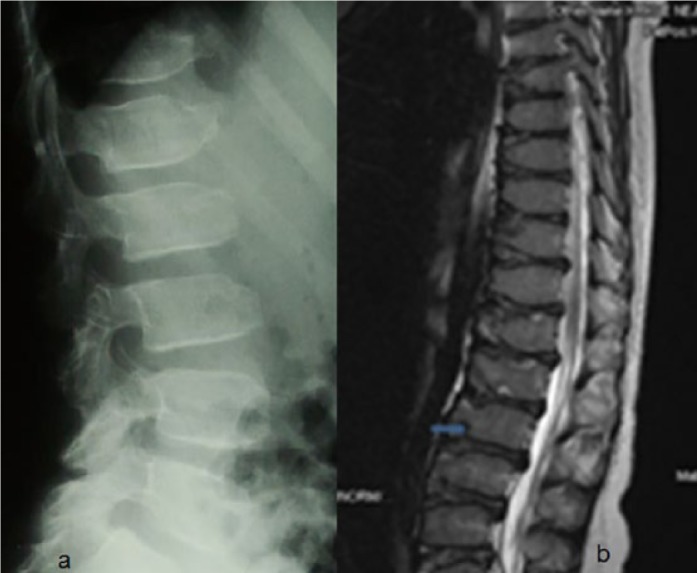
(a) Lateral lumbar spine radiograph in a 9-year-old girl showed defective ossification of the anterior portions of the upper and lower end-plates, and L-3 specifically showed marked anterior ossification defect (anteriorly pointing) associated with reduction of the height of the upper lumbar bodies. (b) Sagittal MRI of the lumbar spine of the same girl at the age of 13 years showed progressive platyspondyly, Schmorl’s node (arrow), anterior end-plates defective ossifications, and signs of osteochondritis (severe irregularities at the end plates of vertebral bodies with intervertebral and retromarginal herniations).

Lateral lumbar spine radiograph in a 9-year-old girl showed defective ossification of the anterior portions of the upper and lower end plates, and L-3 specifically showed marked anterior ossification defect (anteriorly pointing) associated with reduction of the height of the upper lumbar bodies (Figure 1a). Sagittal MRI of the lumbar spine of the same girl at the age of 13 years showed progressive platyspondyly, Schmorl’s node, anterior end-plates defective ossifications, and signs of osteochondritis (severe irregularities at the end plates of vertebral bodies with intervertebral and retromarginal herniations; Figure 1b).

**Figure 2.**
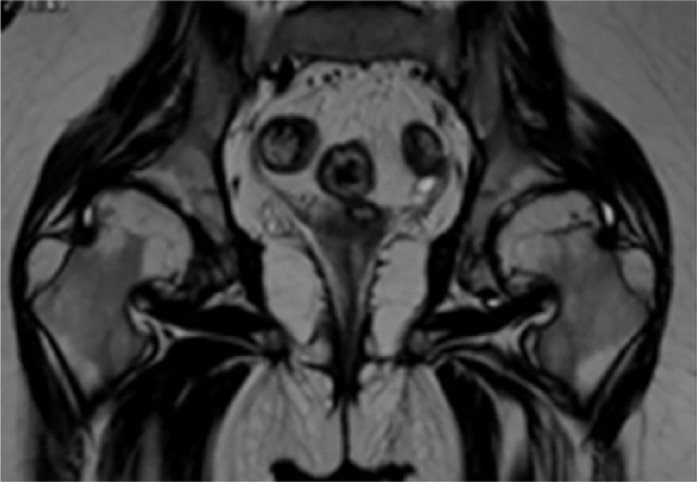
Magnetic resonance imaging (1.5 T) in a 13-year-old girl with PPRC showed intraarticular pathology strongly compatible with the radiographic features of osteoarthritis. Note the laterally prominent femoral head margins create bilateral femoral head asphericity. The capital femoral epiphyses are flattened, irregular associated with metaphyseal fragmentation and irregularity. The superior portions of the acetabular labra were sclerosed with partial detachment.

Pelvic radiographs were scored according to the Kellgren-Lawrence system. Clinical outcomes were assessed by the hip osteoarthritis outcome score. Osteoarthritis (grade III of Kellgren-Lawrence grading scale) signified marginal osteophytes (glans deformity) associated with progressive capital femoral epiphyseal dysplasia and narrowed and irregular joint spaces and coxa vara. MRI (1.5 T) showed intraarticular pathology strongly compatible with the radiographic features of osteoarthritis. Note the laterally prominent femoral head margins create bilateral femoral head asphericity. The capital femoral epiphyses are flattened, irregular associated with metaphyseal fragmentation and irregularity. The superior portions of the acetabular labra were sclerosed with partial detachment (Figure 2).

The genetic testing of PPAC was established, and the diagnosis was confirmed by genetic tests in 2 patients in which a homozygous c.667T>G variation was identified in exon 4 of the WISP3 gene. The third patient was an adult and she was related to one of the patients and she refused genetic tests.

### Group 2

**Figure 3.**
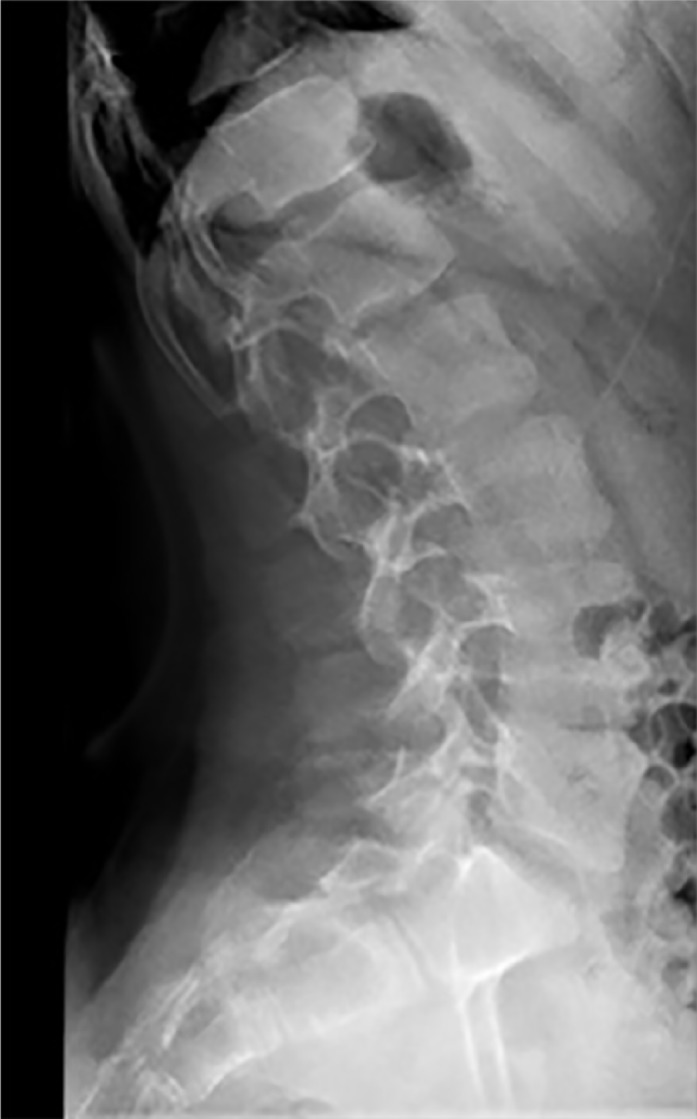
Lateral spine radiograph in a 10-year-old child with Klinefelter syndrome showed exaggerated lumbar lordosis, mild platyspondyly of T11-12, anterior end-plate irregularity of T12, scalloping of the posterior end plate of L3.

**Figure 4.**
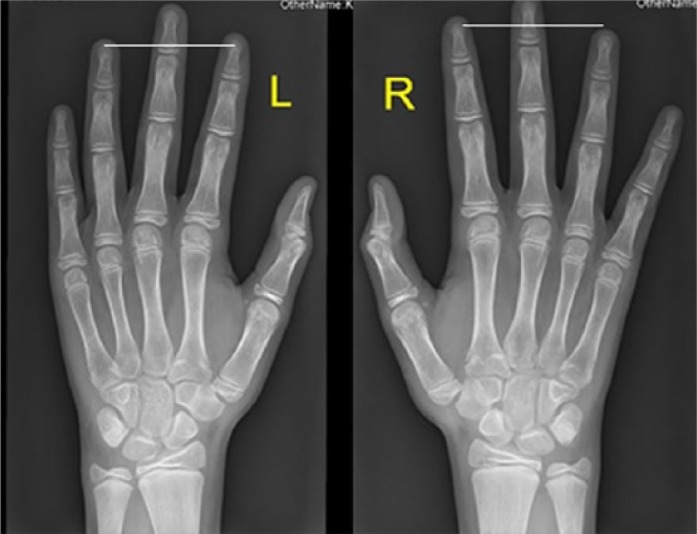
AP hand radiographs in a 13-year-old boy with Klinefelter syndrome showed phalangeal preponderance (normally the length of the fourth metacarpal is equal to length of distal plus proximal phalanges).

Two boys gave the clinical phenotype of Klinefelter syndrome, and on the basis of skeletal survey, the lateral lumbar radiograph showed osteopenia, exaggerated lumbar lordosis, mild platyspondyly of T11-12, anterior end-plate irregularity of T12, scalloping of the posterior end-plate of L3 (Figure 3). AP hands radiographs showed phalangeal preponderance (normally the length of the fourth metacarpal is equal to length of distal plus proximal phalanges) (Figure 4).

Karyotyping confirmed the diagnosis of Klinefelter syndrome and they manifested XXY aneuploidy.

**Figure 5.**
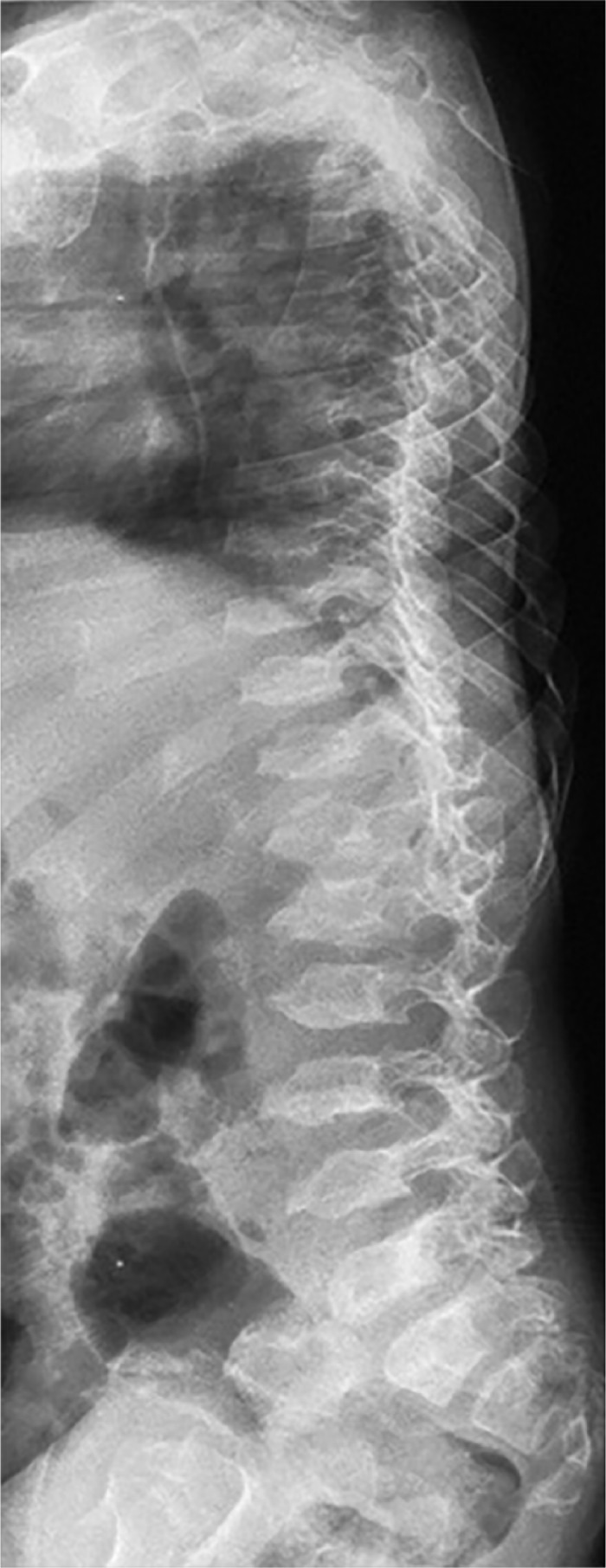
Lateral spine radiograph in a 3-year-old boy with MPS type IVA showed platyspondyly and oval-shaped vertebrae associated with dorsal wedging and deficient ossification of the anterior portions.

One male patient showed the typical radiographic diagnostic criterion of MPS IV. Lateral spine radiograph showed platyspondyly and oval-shaped vertebrae associated with dorsal wedging and deficient ossification of the anterior portions (Figure 5).

Laboratory findings confirmed the diagnosis of MPS IVA and showed increased urinary excretion of keratan-sulfate and chondroitin-6-sulfate. In cultured fibroblasts there was low activity of N-acetylgalactosamine-6-sulfate-sulfatase. Both boys showed missense mutations in the GALNS gene.

## Discussion

Muscular dystrophies are a group of genetically determined, progressive diseases of skeletal muscle. The classification of progressive muscular dystrophy that is most relevant is the system proposed by Walton and Nattrass.^11^

In general, these disorders are present at birth, even though they may not develop their pathological hallmark until later in life, and their diagnosis may therefore be delayed. The clinical features of these disorders are similar, with variations on the theme of weakness, muscle wasting, hypotonia, delayed motor development, and in some joint contractures. There are no pathognomonic clinical features, so that although the diagnosis may be suspected clinically it must be confirmed by muscle biopsy and other sophisticated genetic studies. The presence of dysmorphic facial features, birth defects, or failure to thrive associated with hypotonia may indicate chromosomal abnormality detectable by karyotype.^12,13^

Progressive pseudorheumatoid arthropathy of childhood (PPAC) is a disorder that is characterized by walking difficulties, easy fatigability, and muscular weakness. The overall clinical features are akin to and/or confused with muscular dystrophy and/or juvenile rheumatoid arthritis. The designation pseudorheumatoid points to the deceptive clinical appearance in childhood. In contrast to rheumatic diseases, PPAC is primarily a developmental and not a degenerative disease. The distinguishing features are the presence of platyspondyly and features of bone dysplasia and the absence of destructive bone changes.

Spranger et al^3,4^ gave the name PPAC; they suggested that it is a primary disorder of the joint cartilage. They demonstrated chondral abnormalities in an iliac crest biopsy with nests of clustered chondrocytes with pyknotic nuclei, irregular ground substance, and spiral bundles of thin fiber, mostly without striations. There is no inflammation of the joints and it has been confirmed that the disorder is in fact in connection with noninflammatory chondropathy affecting mainly the articular cartilage. Early onset of cartilage loss is a very important element in the pathogenesis of the disease.

Delague et al^14^ described the molecular study of the WISP3 gene in 9 unrelated consanguineous families originating from the Middle East: 3 from Lebanon, 5 from Syria, and 1 from Palestinian Bedouin descent, all affected with progressive pseudorheumatoid dysplasia. Five different sequence variations were identified in the WISP3 gene, 2 of them being new mutations: the c.589G>C transversion at codon 197, responsible for a splicing defect (A197fsX201); and the c.536_537delGT deletion (C179fsX), both in exon 3. In all other families, the affected patients were homozygous for a previously described nonsense mutation, namely, c.156C>A (p.C52*). The abnormal arrangement of chondrocytes in resting cartilage and growth zones probably reflects the abnormal activity of WISP3, a mesenchymal signaling protein.^14^

Al Kaissi et al^15^ described a male patient with the clinical and radiographic phenotypic characterization of PPAC and manifested a combination of craniocervical and pelvic pathology.

Klinefelter syndrome may remain largely undiagnosed, unless there are certain features suspicious of a chromosomal abnormality such as dysmorphic facial features, birth defects, or failure to thrive. The combination of these features was not encountered in our patients. Most XXY neonates appear normal at birth and abnormal facial or other birth defects are not common manifestations.^16^ Some reports described the phenotypic abnormalities observed in variants of Klinefelter syndrome, including microcephaly, short stature, hypertelorism, flat nasal bridge, clinodactyly, radioulnar synostosis, and genu valgum.^17^ Extreme ligamentous hyperlaxity and muscular wasting have not been reported.

Morquio’s syndromes belong to a family of disorders identified as lysosomal storage diseases, and historically as mucopolysaccharidoses. These disorders are characterized by the lysosomal accumulation of glycoconjugates (glycolipids, glycoproteins, and glycosaminoglycans) due to deficiencies in lysosomal hydrolases responsible for the degradation of these classes of molecules. In Morquio’s syndrome, both mortality and morbidity are related primarily to atlantoaxial subluxation resulting from the instability of odontoid process.^18^

A minor fall or excessive neck extension can result in cord transection and subsequent quadriparesis/death.

In the classic forms of MPS IV, patients usually become symptomatic between 3 and 6 years of age. Generalized joint laxity (a unique feature mostly caused by progressive metaphyseal dysplasia with simultaneous degradation of connective tissues near the joints) was a feature in our patient, making it distinctly different from the other types of mucopolysaccharidoses and/or myopathy.

## Conclusion

The efforts in researching the precise etiology in patients with muscle weakness has not been rewarding as anticipated by many. These groups of children pose real challenges to pediatricians/physicians because muscle weakness may be the misleading clinical feature hiding serious disorders. Children with skeletal disorders are frequently approached with lengthy diagnosis and unnecessary investigations. The clinical and radiographic phenotypes are the baseline tools toward understanding the basis of complex disorders and the guidance for targeted genotype. Sadly, neither the clinical nor the radiological phenotypes are receiving adequate interest and this kind of methodology is rarely practiced. The outcome is prolonged with unfruitful clinical procedures, which exert dreadful emotional and social impact on these children and their families.

The natural history of PPAC is somehow confusing for physicians. The earliest radiographic appearance of spondyloepiphyseal dysplasia is the cornerstone for differentiating PPAC from other musculoskeletal disorders. The boney changes in the spine and the epiphyses are missed by radiologists and rheumatologists, unless adequate experience is available. On the other hand, the diagnostic imaging studies for children with other musculoskeletal system disorders such as Klinefelter or Morquio’s syndromes are deemed useful for management purposes. Finally, we wish to stress that the clinical data need to be analyzed, not in isolation, but with the context of relevant family history, and the targeted/diagnostic molecular diagnostic studies should be based on proper clinical and radiographic phenotypic interpretations.
